# Macroscopic Equations Governing Noisy Spiking Neuronal Populations with Linear Synapses

**DOI:** 10.1371/journal.pone.0078917

**Published:** 2013-11-13

**Authors:** Mathieu N. Galtier, Jonathan Touboul

**Affiliations:** 1 Jacobs University, Bremen, Germany; 2 The Mathematical Neuroscience Laboratory, CIRB/Collège de France, CNRS UMR 7241, INSERM U1050, UPMC ED 158, MEMOLIFE PSL, Paris, France; 3 BANG Laboratory, INRIA Rocquencourt, Paris, France; University of Namur, Belgium

## Abstract

Deriving tractable reduced equations of biological neural networks capturing the macroscopic dynamics of sub-populations of neurons has been a longstanding problem in computational neuroscience. In this paper, we propose a reduction of large-scale multi-population stochastic networks based on the mean-field theory. We derive, for a wide class of spiking neuron models, a system of differential equations of the type of the usual Wilson-Cowan systems describing the macroscopic activity of populations, under the assumption that synaptic integration is linear with random coefficients. Our reduction involves one unknown function, the effective non-linearity of the network of populations, which can be analytically determined in simple cases, and numerically computed in general. This function depends on the underlying properties of the cells, and in particular the noise level. Appropriate parameters and functions involved in the reduction are given for different models of neurons: McKean, Fitzhugh-Nagumo and Hodgkin-Huxley models. Simulations of the reduced model show a precise agreement with the macroscopic dynamics of the networks for the first two models.

## Introduction

The activity of the brain is characterized by large-scale macroscopic states resulting from the structured interaction of a very large number of neurons. These macroscopic states correspond to signals experimentally measured through usual recording techniques such as extracellular electrodes, optical imaging, electro- or magneto- encephalography and magnetic resonance imaging. All these experimental imaging protocols indeed record the activity of large scale neuronal areas involving thousands to millions of cells. At the cellular level, neurons composing these columns manifest highly complex, excitable behaviors characterized by the intense presence of noise. Several relevant brain states and functions rely on the coordinated behaviors of large neural assemblies, and resulting collective phenomena recently raised the interest of physiologists and computational neuroscientists, among which we shall cite the rapid complex answers to specific stimuli [1], decorrelated activity citeecker-berens-etal:10,renart-de-la-rocha-etal:10, large scale oscillations [2], synchronization [3], and spatio-temporal pattern formation [4], [5].

This motivates the development of models of the collective dynamics of neuronal populations, that are simple enough to be mathematically analyzed or efficiently simulated. A particularly important problem would be to derive tractable macroscopic limits of the widely accepted and accurate Hodgkin-Huxley model [6]. However, describing the activity of a network at the cellular scale yields extremely complex, very high dimensional equations that are mathematically intractable and lead to excessively complex and time consuming numerical simulations. Such simulations of large-scale systems have been reported in [7]. In that study, the author performs a numerical simulation of a network composed of one hundred billion neurons (the order of magnitude of a macroscopic brain area of 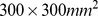) and one quadrillion synapses, based on a simplified nonlinear integrate-and-fire neuron. The simulation of the activity of one second of the network took 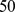 days on efficient machines back in 2005. Although machines have become faster, taking into account more biologically plausible neuronal models in detailed microscopic simulations takes even more time [8], [9], and developing a supercomputer-based simulations of the brain at a cellular level is an important endeavor currently undertaken [10]. The tenet of the present manuscript is precisely that theoretical approaches may allow rigorously deriving macroscopic models that can be efficiently implemented and which reproduce accurately the dynamics of large networks.

The question of the macroscopic modeling of cortical activity and their relationship with microscopic (cellular) behavior has been the subject of extensive work. Most studies rely on heuristic models (or firing-rate models) since the seminal works of Wilson, Cowan and Amari [11], [12]. These models describe a macroscopic variable, the population-averaged firing-rate, through deterministic integro-differential or ordinary differential equations. Analytical and numerical explorations characterized successfully a number of phenomena, among which spatio-temporal pattern formation and visual illusions (see [13] for a recent review). This approach was complemented by a number of computational studies introducing noise at the level of microscopic equations, the effect of which vanishes in the limit where the number of neurons tends to infinity. These approaches are generally based on simplified neuron models and make significant assumptions on the dynamics (e.g. sparse connectivity [14], Markovian modeling of the firing and van Kampen expansion [15]). Relationship between spiking neuronal networks and mean firing rates in simplified models and deterministic settings has also been the subject of a number of outstanding works [16], [17]. These averaging techniques were based on temporal averaging of periodic spiking behaviors. For instance, in [17], the author presents a reduction to Wilson-Cowan systems for the single-cell deterministic Morris-Lecar system, taking advantage of the separation of timescales between slow synapses and cell dynamics. In contrast with these researches, we propose a mixed population and temporal averaging for stochastic networks, taking advantage of the collective effects arising in large networks.

Despite these efforts, deriving the equations of macroscopic behaviors of large neuronal networks from relevant descriptions of the dynamics of noisy neuronal networks remains today one of the main challenges in computational neuroscience, as discussed in P. Bressloff's review [13]. In the present manuscript, we contribute to this axis of research with a hybrid theoretical-computational approach. Necessarily, our rigorous approach will impose two main assumptions. First, synapses are assumed to be linear exponential filters. This assumption, although usually made in the reduction of spiking network into rate-based networks (see [18], chapter 11), disregard an important feature of chemical synapses: a threshold non-linearity. This non-linearity, albeit weak, induces a significant increase in the complexity of the microscopic equations. Thus, this assumption will simplify the underlying mathematical problem and make it possible to focus on the neuronal excitability. Besides, note that the approach is particularly well fitted to networks connected through gap-junctions (electrical synapses), since these are well described by linear interactions [19], [20]. Second, the strength of connections between neurons is chosen random and independent. This hypothesis allows to take into account heterogeneities of the synapses, and avoiding to impose specific connectivity patterns. Inspired by statistical mechanics methods, we start from rigorously derived limits of neuronal network equations [21] with excitable dynamics of Hodgkin-Huxley type. The resulting equations, referred to as the *mean-field equations*, are hard to interpret and to relate to physical observable quantities. In the gas dynamics domain, mean-field equations such as Boltzmann's equation were used to derive the behavior of macroscopic quantities such as the local density, macroscopic local velocity and local temperature fields, in relationship with the microscopic activity of the particles, and resulted in the derivation of the celebrated Navier-Stokes equations that provide important information on the fluid dynamics. In our biological case, a particularly important quantity accessible through measurement is the macroscopic variable corresponding to an averaged value, over neurons at certain spatial locations, and on a specific time interval, of the activity of each cell. We will therefore aim at describing this variable in order to reduce the complex high dimensional noisy dynamics of microscopic descriptions of neural networks into a simple, deterministic equation on macroscopic observables.

As opposed to a large body of literature [14], [22]–[24], this paper does not aim at computing the firing rate function of the network. It rather aims at deriving a dynamical system describing the macroscopic activity of the network, in the spirit of [25] where the authors derive partial integro-differential equations describing the population density of a network of integrate and fire neurons. In contrast, our approach will consist in computing an effective non-linearity function (slightly different from the firing rate) involved in the macroscopic equations.

The paper is organized as follows. In the Material and Methods section, we will introduce the basic network equations and their mean-field limits, and describe the methodology we propose for deriving macroscopic equations. We first show a rigorous derivation for the deterministic McKean neurons and numerically extend this to more general cases. This method reduces the dynamics of the average firing-rate to the knowledge of a particular function, the effective non-linearity, which can be numerically computed in all cases. This methodology is put in good use in the Results section in the case of the McKean, Fitzhugh-Nagumo and Hodgkin-Huxley neurons. In each case, the effective nonlinearity is numerically computed for different noise levels. The reduced low-dimensional macroscopic system is then compared to simulations of large networks, and will show a precise agreement. We also numerically investigate the robustness of the reduction with respect to the variation of parameters. The discussion section explores some implications of the present approach.

## Materials and Methods

In this section we introduce the networks models considered, their mean-field limits and the formal derivation of the dynamics of averaged firing-rate models. This approach will be used in the result section to derive macroscopic limits and demonstrate the validity of the reduction. The python programs corresponding to the simulations can be downloaded at the url https://www.rocq.inria.fr/bang/JT/Documents/spatav_galtier_touboul.zip.

### Neurons and networks

We consider a network of P populations being composed of 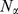 neurons for population 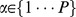. Motivated by the large number of neurons involved in each populations at functional scales, we will be interested in the limit where all 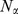 tend to infinity (the *mean-field limit*) in order to take advantage of possible regularization and averaging effects. Each neuron 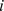 in population 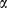 is described by the membrane potential 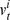 (also called activity of a neuron in this paper) and additional variables gathered in a 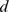-dimensional variable 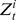, representing for instance ionic concentrations in the Hodgkin-Huxley model, or a recovery variable in the Fitzhugh-Nagumo or McKean models. These variables satisfy a stochastic differential equation:
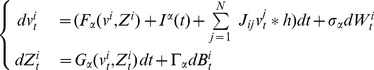
(1)


In this equation, the functions 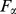 and 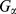 describe the intrinsic dynamics of all the neurons in population 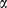. The parameters 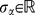 and 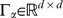 describe the intensity of the noise, classically driven by independent one (resp. 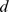) dimensional Gaussian white noise 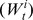 (resp. 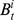). The functions 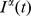 represent the external input received by neurons of population 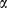. The coefficients 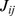 are the synaptic weights of the connection from neuron 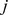 to neuron 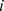. Spiking interactions between neurons are here modeled as the convolution of the presynaptic membrane potential with the impulse response of the synapse noted 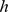, where 
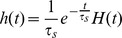
 with 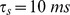 is the characteristic time of the synapses and 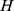 the Heaviside function. Therefore, this model assumes linear synaptic integration.

Three main models used in computational neuroscience addressed in the present manuscript are the McKean model [26], the Fitzhugh-Nagumo model [27] and the Hodgkin-Huxley model [6]. These are parametrized so that the time unit is one millisecond.

#### The Hodgkin-Huxley model

is probably the most widely accepted neuron model from the electrophysiological viewpoint. The Hodgkin-Huxley model describes the evolution of the membrane potential in relationship with the dynamics of ionic currents flowing across the cellular membrane of the neuron. It was introduced in the 1950s in [6] after thorough observation of the giant squid axon revealed the prominent role of potassium and sodium channels for excitability, and leak chloride currents. Networks of Hodgkin-Huxley neurons are described by the stochastic differential equation:
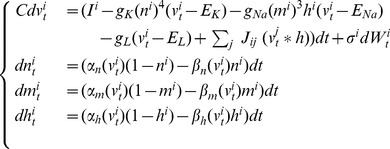
(2)where



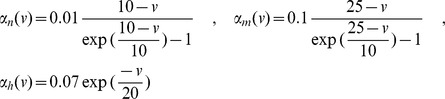


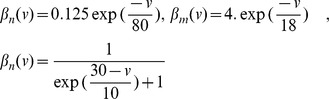
and 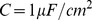, 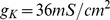, 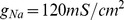, 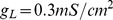, 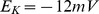, 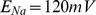 and 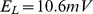. These parameters correspond to a resting state of the membrane potential 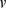 equal to 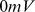 (as opposed to McKean and Fitzhugh-Nagumo models which have a negative resting state). The dynamics of this system shows deep non-linear intricacies even in the case of deterministic, single-neuron system.

#### The Fitzhugh-Nagumo model

was introduced in [27] as a simple model reproducing the essential features of the Hodgkin-Huxley model. It has been widely studied as a paradigmatic low-dimensional excitable systems which produces a wide range of spiking behaviors. The Fitzhugh-Nagumo model describes the activity of the membrane potential 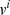 and recovery variable 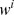 of neuron 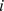 in the network through the equations:
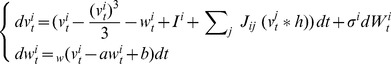
(3)with 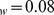, 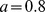 and 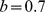.

#### The McKean model

is a piecewise continuous approximation of the Fitzhugh-Nagumo model that presents the mathematical advantage of allowing explicit calculations and analytic developments [20], [26]. In that model, the membrane potential of neuron 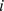, denoted 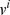, is coupled to an adaptation variable 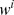 and to the membrane potential of other neurons, and satisfy the equations:
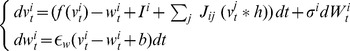
(4)


with
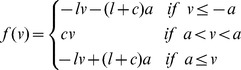
and 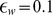, 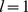, 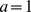, 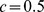, and 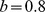.

### Mean-field limits

The behavior of very large random stochastic networks can be adequately described in the *mean-field limit* corresponding to the asymptotic regime where the number of neurons goes to infinity [21] (the finite-size error is explicitly characterized as the distance between the finite-sized network and the limit, and vanishes as 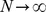). Remarkably, in that limit, each neuron is an independent realization of the same stochastic process described by an implicit stochastic equation (called McKean-Vlasov equation, or simply the mean-field equation). We review in this part the mean-field theory and apply it to our cases for a network of populations of neurons described by (1).

In our model, we assume that the synaptic weights are randomly drawn from a normal law. Specifically, we consider that the connection 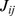 between neurons 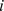 and 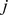 belonging to populations 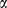 and 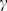 respectively is a Gaussian random variable with mean and standard deviation depending on the populations they belong to:
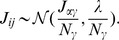




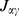
 is the averaged connectivity weight between populations 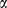 and 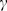, and 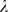 quantifies the heterogeneity (disorder) of these synaptic weights.

As shown in [21], [28], in the limit where all 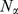 go to infinity, all neurons belonging to the same population (say, 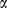) are independent and have the same probability distribution solution of the mean-field equation:
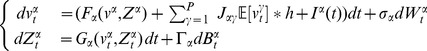
(5)


The independence property (called propagation of chaos in Boltzmann's kinetic theory) ensure that in the limit, each neuron produces an independent realization of the same probability distribution, and thus, samples this law. Therefore, any statistics of the neuronal activity in a population can be accessed. An important example is the empirical average of the activity, which converges towards the expectation of the solution to the mean-field equation. This property has the important consequence that the averaged activity of all neurons in a population can be accessible through the mean-field equations.

Let us eventually notice that the mean-field equation (5) involves an interaction term in the form of the expectation of the activity (solution of the equation). In that sense, this is not a standard stochastic differential equation, but an implicit (fixed-point) equation in the space of stochastic processes. The mathematical study of this type of equation is generally extremely complex. In our present approach, we will manage to bypass this difficulty by introducing a new quantity representing the macroscopic activity of a population.

### Firing Rates, Macroscopic Activity and Dynamics

Now that we introduced the network models and the limits we are interested in, we are in a position to define the observable macroscopic quantity that will describe the activity of the network.

The averaged firing-rate is usually considered as a relevant macroscopic description of the population activity. Heuristically, this quantity corresponds to the number of spikes fired in a certain time window averaged over all neurons in the same population. Of course, counting discrete events is a non-trivial operation, and several computational definitions have been proposed [29].

This complexity motivates the introduction of an analogous variable to the firing rate, which we call macroscopic activity, simply defined as the averaged membrane potential of neurons belonging to a given population and within a certain time window. Although this measure does not explicit count spikes, it is closely related to the firing-rate. Indeed, given that neurons communicate via spikes which are stereotyped electrical impulses of extreme amplitude, averaging the value of the membrane potential during a time window and dividing by the area under a spike provide a rough estimate of the number of spikes emitted. The main difference between the two measures is that it is affected by the subthreshold neuronal activity [30], [31] which does not intervenes in the computation of firing rates. However, this definition has the mathematical interest of being a linear transformation of the activity of neurons.

In detail, we define the *macroscopic activity* 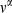 of population 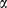 as the averaged membrane potential 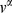 over the neurons in the populations temporally convolved with the time window function 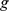 of width 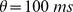 larger than the duration of a spike, but small enough to resolve fine temporal structure of the network activity. The time window is defined as 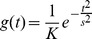 with 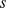 chosen so that 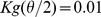 and 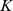 so that 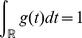. The property of neurons to have independent voltages in the large size limit allows us to identify the population-averaged voltage with the statistical expectation of the voltage variable in the mean-field limit. This leads to the following expression for the macroscopic activity:
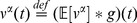
(6)


Equation (5) therefore allows to characterize the macroscopic activity as the solution of the equation
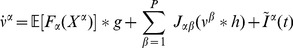
(7)where 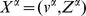 and 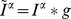.

This equation is not closed because of the term 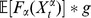 which is not expressed as a function of the macroscopic activity. Because of the nonlinearity of 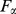, it is not likely that this quantity only depends on 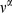. Moreover, the membrane potential depends on additional variables and the macroscopic activity hence involves expected value of functions of these variables convolved with the time kernel 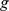. We now show how to reduce these equations to a closed system on the variables 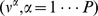.

### Rigorous derivation for deterministic McKean neuron

In order to explain the principle of the reduction, we start by treating analytically the simplest case considered, namely the McKean neurons networks with no noise. In this model, we compute 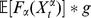 in a closed form. This leads to a rigorous derivation of the reduced model.

We consider a McKean network (4) in the mean field limit (5) and further consider 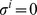. The equations of the system reduce to:
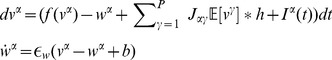
(8)


In the sequel, we use an implicit integration of the adaptation variable 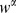 in (8) and replace in the voltage equation, the adaptation variable by its expression as a function of voltage: 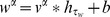, where 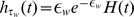. Using the commutativity of the convolution and equation (6), we obtain the exact macroscopic equation for the McKean neuron:
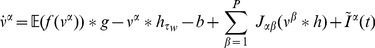
(9)


**Figure 1:**
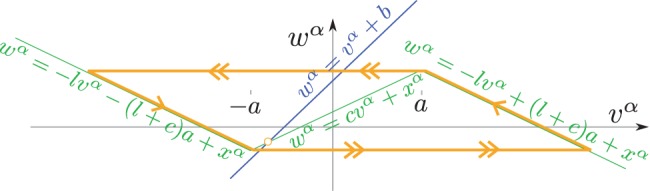
Phase plane of the deterministic McKean neuron. Corresponds to equations (8) with 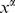 a constant. When the blue line and any of the decreasing green lines intersect, then there is a stable fixed point. When the blue curve intersects the increasing green line, then there is a periodic orbit (this is the case shown here). The periodic orbit corresponds to the non-smooth orange trajectory composed of two branches on the slow manifold (single-arrowed segments) and horizontal double-arrowed segments correspond to the fast transitions.

As said before, the only unknown term in the formula above is 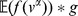. In order to perform our reduction, we use the assumption that the adaptation variable and input are slow, which allows an adiabatic reduction, i.e. allows to consider 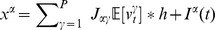 as a constant, i.e. 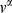 reaches its equilibrium value very fast (we will numerically show in the sequel that the reduction is quite robust when the assumption is not perfectly satisfied). In this approximation, 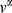 can be considered uncoupled from the others populations and it has the same dynamics as a single McKean neuron, whose phase plane is shown in Figure 1.

Under the assumption that the recovery variable is very slow (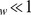), the state of neurons in population 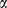 is essentially projected on one of the two slow manifold, corresponding in the phase plane Figure 1 to the single-arrowed orange branches of the 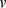-nullcline. Fast switches between these two branches of the slow manifold occur when the trajectories reach an extremity of the manifold. Except during the very fast transitions, it holds that 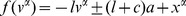 and hence
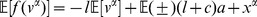



with 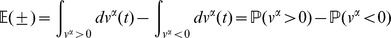. Therefore we have:
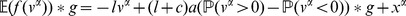



We write 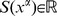 the value toward which the function 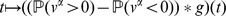 converges when the McKean is stimulated by a constant input 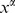 (thus we discard the initial transient). Computing 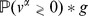 for a constant effective input 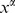 amounts to computing the proportion of time system a McKean neuron spends on (or close to) the slow manifolds 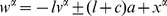. This can be performed analytically. Indeed, there are two different cases:

- If 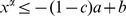 (resp. 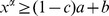).

Then the system has a single stable fixed point on the negative (resp. positive) slow manifold. In Figure 1, this corresponds to the blue curve crossing the green piecewise cubic where the latter is decreasing. In this case, 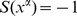 (resp. 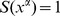).

- If 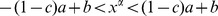.

Then the system is oscillating on a deterministic limit cycle represented in orange in Figure 1. In this case, 
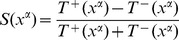
 where 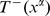 (resp. 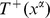) is the duration it takes for the system to go along the negative (resp. positive) part of the slow manifold. Following [32], we can access these values. Indeed, assume the fast membrane potential immediately goes to one of the slow nullclines. This gives the equation: 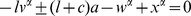. Injecting this in the slow equation and integrating along relaxation orbit (orange path in Figure 1) leads to
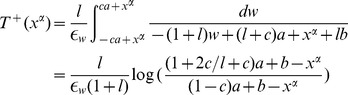



Similarly,
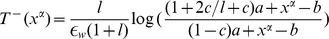



Therefore, for 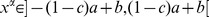


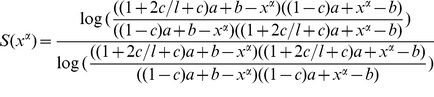
(10)


**Figure 2:**
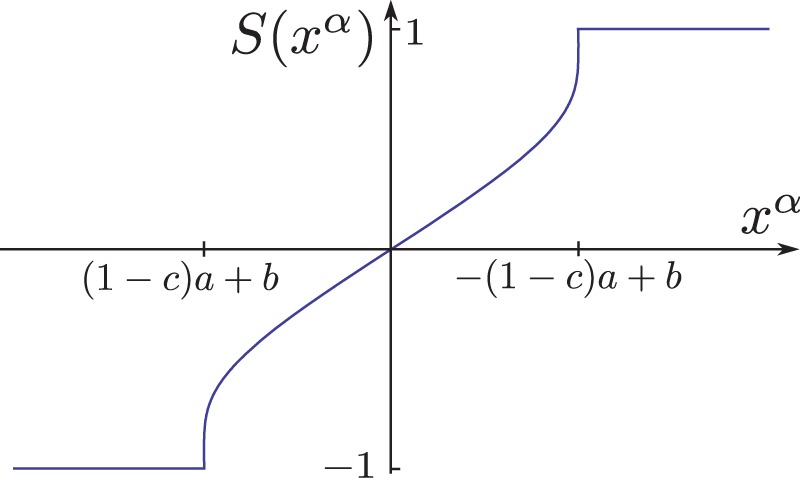
Function 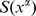 for the deterministic McKean model given in equation 10.

This function is shown in Figure 2. It is a non-smooth sigmoidal function with vertical tangents at 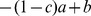 and 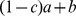. This corresponds to the transition from a fixed point to the oscillatory pattern.

Based on equations (9) and the definition of the sigmoid (10), we are now in position to define an averaged model describing the evolution of the macroscopic population activity. It takes the form of a self consistent, non autonomous, delayed differential system:
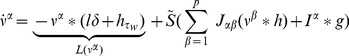
(11)where 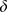 is the Dirac function and 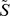 is an element-wise function such that 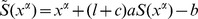. Note that this function provides an analytic expression for the bifurcation diagram shown latter.

### Numerical computation the effective non-linearity for any neuron model

In the general case (stochastic nonlinear neurons), one can numerically compute the effective non-linearity. To make sense of the term 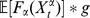, we introduce the ansatz that this term can be written as the sum of a linear functional of the macroscopic activity and a non-linear term applied to the effective input to a population (including the synaptic connections). Defining this effective input 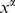 as:
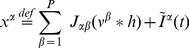
(12)


the ansatz reads:
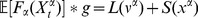
(13)where 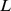 is a linear functional and 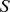 is a non-linear mapping which remains to be determined. The choice of this ansatz was motivated by the analytical treatment of networks of deterministic McKean neurons, which naturally exhibits a relation like (13). The choice of the linear functional 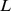 is dictated by the neuron model used.

To evaluate 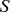, one need to assume that both the inputs and the synapses are slow compared to the dynamics of the neurons. Thus, the effective input 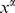 can be considered as constant during the time the neurons reach an asymptotic regime related to that input state. If this regime is stationary, the value function 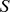 at 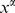 will be the average of 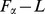 applied to that stationary stochastic process, and therefore will provide a quantity only depending on 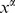. If the regime is periodic in law, then taking a time window 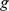 of size 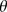 larger than the period will also yield a constant value for our nonlinear term. Because the mean-field equation (5) and a single neuron equation only differ in the interaction term which is assumed constant here, the computation of 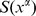 simply corresponds to computing the temporal average of 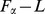 along the trajectory a single noisy neuron forced with a constant input 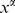. Actually, for readability reasons, after combining equations (7), (12) and (13) into a final macroscopic equation, we will rather focus on the function 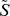 which is simply defined as 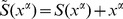.

**Figure 3:**
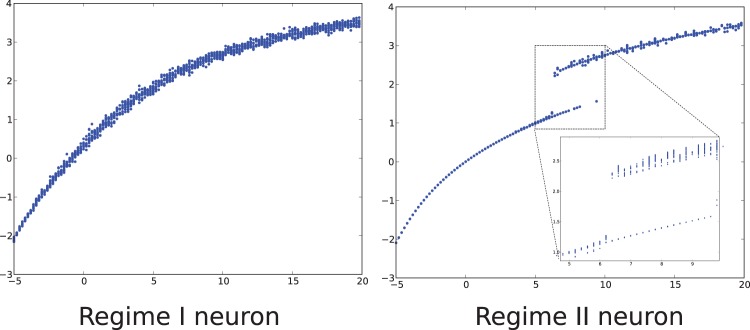
Effective nonlinearity of regime I and II neurons. Value of 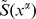 computed for 10 different initial conditions (and 200 in the inset of the right picture) for the Hodgkin-Huxley model with noise 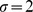 (left) and 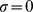 (right). This shows that the level of intrinsic noise change the regime of a neuron.

One of the pitfalls of this methods occurs if the mean-field equation present multiple stable stationary or periodic attractors. In that particular case, the quantity 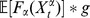 can take different values depending on the initial condition. A neuron model (together with a particular set of parameters) will be said to be of regime I if there is only one of these attractor for any initial condition. Similarly, if there are p-attractors, the neuron model is said to be in regime p. Figure 3 shows different values for 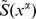 when starting from different initial condition. Figure 3 left shows the solution for regime I and Figure 3 right for regime II. The points obtained are then segmented into a few clusters (in our case, one or two in regimes I and II respectively) and smoothed out into a surface (or a union of surfaces) see Figure 5. This procedure is relatively time consuming. The result of extensive simulations on the McKean, Fitzhugh-Nagumo and Hodgkin-Huxley models, using this numerical procedure, are freely available online as well as the algorithm generating these data.

### Linear part 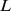 for the different models

Identifying 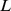 for a given model, consists in gathering the most linear terms in the intrinsic dynamics of a single neuron 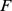. This linear term can be time-delayed. The linear part of each of the considered neurons are shown in table 1.

**Table 1:** Linear part *L* for the different models.

Model	Linear part
McKean	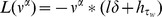
Fitzhugh-Nagumo	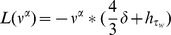
Hodgkin-Huxley	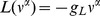

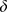
 is the Dirac function centered at 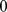 and 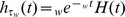 for the first two models (where 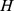 is the Heaviside function).

For McKean and Fitzhugh-Nagumo models, the convolution accounts entirely for the existence of the adaptation variable 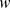 which will therefore still be present in the reduced model. The choice of the linear part of the McKean model naturally comes out of the computations. The choice of the coefficient 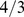 for the Fitzhugh-Nagumo model relies on an analogy to the McKean neuron. It corresponds to the (absolute value) of the slope of the straight line approximating the negative decreasing part of the non-linearity Fitzhugh-Nagumo 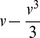. As of the Hodgkin-Huxley, we were not able to extract a linear delayed term in the intrinsic dynamics of a neuron so we simply chose the linear decay naturally present in the equations.

### Simulation of the macroscopic equations

For regime I neurons, when the effective non-linearity 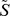 is univalued, simulations of the macroscopic equations simply reduce to solving numerically the following ordinary differential equation
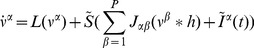
(14)


For regime II neurons, when the initial condition is not in the bistable region, we will consider that the averaged system pursues on the initial attractor (fixed point or spiking cycle) when possible, and switches attractors if the activity brings the system in regions where the initial attractor disappears. In details, let us denote by 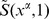 the branch of stable fixed points, defined as long as 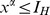, and by 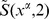 the branch of periodic orbits defined for 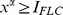. The macroscopic activity of population 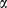 in a 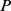-population network hence satisfies the equations:
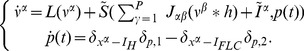
(15)


This approximation will be efficient if the probability to switch from one attractor to the other is small, e.g. for small noise.

## Results

In this section, we evaluate the accuracy of the reduced model presented above for the three neuron models McKean, Fitzhugh-Nagumo and Hodgkin-Huxley. First, we address the computation of the effective non-linearity both in the deterministic and noisy cases. Second, we confront the time course of the macroscopic activity calculated according to our reduction against the a posteriori average of the activity of a spiking network.

### Effective non-linearity without noise

**Figure 4:**
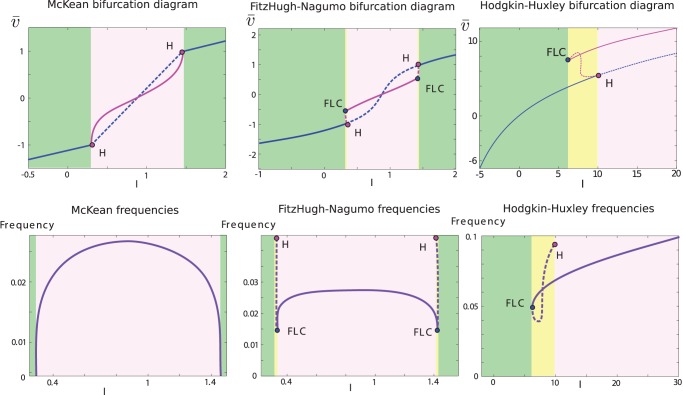
Bifurcation diagrams of single neurons as a function of the input 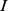. The upper row shows the temporal average of the solutions (i.e. the fixed points and average value in the case of periodic orbits) and the lower row shows the frequency of the regular spiking regime, in the McKean model (left), Fitzhugh-Nagumo model (center) and Hodgkin-Huxley model (right).

In the deterministic reduction (small noise limit), the effective non-linearity can be obtained through the bifurcation analysis of a single neuron. Indeed, the effective sigmoid amounts to computing the temporal average 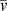 of the voltage solution of the deterministic single-cell system upon variation of the input. The result of that analysis using the numerical software XPPAut [33] is displayed in Figure 4. In these diagrams, we colored regions of stationary solutions (green), periodic solutions (purple) and bistable regimes with co-existence of a stable stationary solution and a stable periodic orbit.

#### The McKean neuron

Although the deterministic McKean neuron has been analytically treated previously, we now consider it under the angle of bifurcations for consistency with the other models. In the McKean neuron, the non-differentiable, piecewise-continuous nature of the flow gives rise to a non-smooth Hopf bifurcation associated with a branch of stable limit cycles. The emergence of the cycle arises through a non-smooth homoclinic bifurcation, hence corresponding to the existence of arbitrarily slow periodic orbits, typical of a class I excitability in the Hodgkin classification. In this model, an important distinction is the absence of bistable regime: the average variable has a unique value whatever the initial condition and whatever the input chosen. In the present case, stable permanent regimes are unique. Therefore, there exists a single-valued function 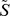 defining the dynamics of the macroscopic activity through equation (14).

#### The Fitzhugh-Nagumo model

The bifurcation diagram of the Fitzhugh-Nagumo neuron as a function of the input level (see Figure 4 middle column) presents a very small parameter region of multi-stability. For small negative input, the system presents a stable fixed point. Increasing the value of the input makes the fixed point lose stability through sub-critical Hopf bifurcation, and unstable limit cycles appear. These limit cycles undergo a fold and a branch of limit cycles appear, overlapping in a small parameter region the state where a stable equilibrium exists. This small parameter region again corresponds to a bistable regime with co-existence of a resting state and of a regular spiking behavior. This branch of stable limit cycles corresponds to a regular spiking regime. As the input is further increased (in a biologically unplausible range), the same scenario arises symmetrically: the branch of stable periodic orbits undergoes a fold of limit cycles, a branch of unstable periodic orbits emerges from this bifurcation and connects with the unstable fixed point at a sub-critical Hopf bifurcation and the unstable fixed point gains stability. Here again, the neuron corresponds to a class II excitability and a regime II, but the system could be well approximated by a excitability I/regime I since the bi-stability region is of very small extent and the periodic orbits have very small periods when appearing, close from a class I excitability. Thus, we consider that the macroscopic activity evolves according to equation (14) as illustrated in the following.

#### The Hodgkin-Huxley model

This model displays qualitatively the same features as that of the Fitzhugh-Nagumo model but with significant quantitative differences. In particular, the bifurcation diagram of the Hodgkin-Huxley neuron (Figure 4 right column) displays multi-stability over a larger range of input values. The bifurcation diagram displays a branch of stable fixed points (green region) that undergo a sub-critical Hopf bifurcation for 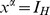, associated to a family of unstable limit cycles (pink dotted lines represent the average value of 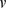 along the cycle). This family of unstable limit cycles connects with a branch of stable limit cycles (pink solid line) through a fold of limit cycles bifurcation. These stable limit cycles are the unique attractor for large input (implausibly large input values will nevertheless see these cycles disappear in favor of a high voltage fixed point). The system presents a bistable regime (yellow input region) where a stable fixed point and a stable periodic orbit co-exist. The frequency along the cycle (Figure 4 right column) shows a class II excitability in the Hodgkin classification: oscillations appear with a finite period and a non-zero frequency. The hysteresis present in the yellow region corresponds to what we called a regime II. In simulations, when the initial condition is not in the bistable region, we will consider that the system pursues on the initial attractor (fixed point or spiking cycle) when possible, and switches attractors if the activity brings the system in regions where the initial attractor disappears, as explained in the Material and Methods section. This method is chosen here because the bi-stability only appears for small noise, regimes in which switches between the different attractors are rare.

### Effective non-linearity with noise

**Figure 5:**
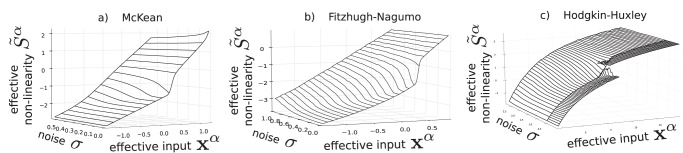
Effective non-linearities surfaces in the McKean, Fitzhugh-Nagumo and Hodgkin-Huxley model. Observe that noise tends to have a smoothing effect on the sigmoids.For the Hodgkin-Huxley model, we have empirically chosen a noise threshold under which the neuron was considered regime II and above which it is regime I. There are thus 2 branches below the threshold and only one above.

In order to compute effective nonlinearities in the presence of noise, we resort to numerical simulations. The method described in Material and Methods provide the surfaces plotted in Figure 5. In that picture, the effective non-linearity is displayed as a surface, plotted for fixed parameters except noise intensity 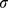 and effective input 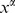. It appears relatively clear in the figure that noise tends to have a smoothing effect on the sigmoids. This effect is particularly clear in the Hodgkin-Huxley model where a multivalued function (regime II) is turned into a single valued smooth function (regime I).

In the case of the Fitzhugh-Nagumo model, we observe that the regime II is not observed in simulations in the presence of noise. This is due to the smallness of the parameter region corresponding to the bistable regime, and the averaged system can be well approximated by regime I dynamics. In the case of the Hodgkin-Huxley network, there are clearly two different behaviors depending of the level of intrinsic noise. When the noise is small (resp. large) the neuron is regime II (resp. I). Interestingly, this shows how a strong noise can qualitatively simplify the macroscopic dynamics of a network.

### Comparison between reduced model and averaged spiking network

We now simulate large networks of McKean, Fitzhugh-Nagumo and Hodgkin-Huxley neurons, compute numerically their macroscopic averaged activity, and compare the dynamics of this variable to simulations of the reduced ordinary differential equation involving our effective non-linearity function in all three cases.

**Figure 6:**
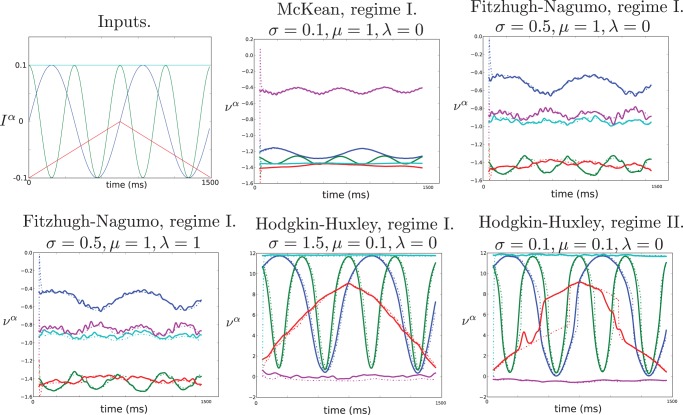
Comparison between the network simulations and computation of the averaged macroscopic variables (plain lines) and simulations of the macroscopic equations (dashed lines). Averaged macroscopic variables are in plain lines and simulations of the macroscopic equations are in dashed lines. The variable related to the 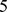 distinct populations (see text) are depicted in different colors. The inputs 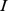 to the McKean and Fitzhugh-Nagumo networks are shown in (a), and for Hodgkin-Huxley networks we took an affine transform of these curves: 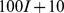. Transient phases in which the averaged microscopic system is imprecise due to the convolution with the symmetric window 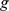 are not plotted. Initial mismatch is due to different initial conditions for both systems. We can observe it quickly disappear, showing the robustness of the reduction to variations of the initial conditions. The simulations where done using a stochastic Euler algorithm with 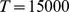 (resp. 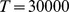) time steps of size 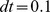 (resp. 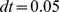) for McKean and FitzHugh-Nagumo (resp. Hodgkin-Huxley) networks.

We consider network models with 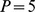 populations, each made of 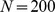 neurons, with averaged connectivity weight 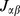 chosen randomly according to a normal law: 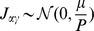. The neuron 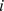 in population 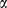 receives an input 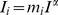, where 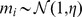. The mean-field theory [21] works if 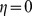, i.e. all the neurons in a population receive the same input. Therefore, the results in this section correspond to 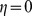. However, the robustness of the approximation to variation of 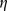 will be tested in the next section. Some parameters are constant for all simulations: 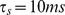, 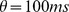. Simulation dependent parameters are detailed in the caption of Figure 6 which gathers the comparison for the different models.

The comparison for the McKean and Fitzhugh-Nagumo neurons show a precise match see Figure 6 (top middle and right, and bottom left) even when the strength 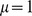 of the connections is strong enough to significantly modify the shape of the input signal.

The comparison for the Hodgkin-Huxley neuron are only a relative success. The first difficulty arises, as expected, for small noise within regime II where the reduction is not univocally determined. The algorithm proposed in Material and Methods to simulate the regime II networks fails reproducing faithfully the averaged spiking network, see Figure 6 bottom right. The network activity is precisely recovered for input that cross relatively rapidly the multistable region (blue and green curve) or that do not intersect the multivalued input region (cyan and purple). However, the red population, spending much time in the multivalued region is not well approximated by the macroscopic activity model: the network equations may randomly switch from spike to rest, which produces the irregular macroscopic activity, whereas the smooth firing rate does not show such switches. These switches arise randomly, and vary from realization to realization. Notwithstanding these unavoidable errors, we observe a fair fit of the macroscopic activity model, which recovers most of the qualitative properties of the network activity. Eventually, we note that even in this configuration, the reduced model is remains accurate when the connections between populations are kept small, i.e. 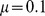 in Figure 6 bottom middle.

### Robustness to parameter change

**Figure 7:**
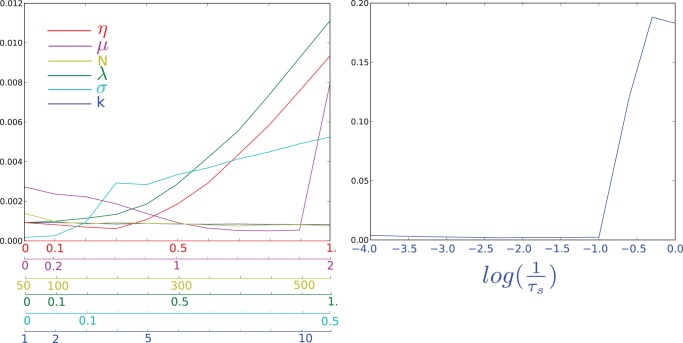
Robustness of the reduction with respect to the variation of the parameters. The y-axis corresponds to the mean of the absolute value of the difference between the a posteriori averaged full system and the reduced system. (left) 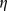 corresponds to the heterogeneity of the inputs to each population. 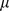 corresponds to the strength of the connections between populations. 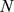 is the number of neurons per population. 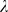 corresponds to the heterogeneity of connections within populations. 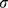 is the intrinsic dynamic noise added to each neuron. 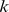 is an index of the input speed, it corresponds to feeding the network with 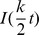 where 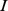 is the function displayed in Figure 6 top left. 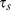 (in ms) is the characteristic time of the synapses.

In Figure 7, we illustrate the robustness of the reduction with respect to the variation of some parameters for the McKean neurons. These results are very similar for Fitzhugh-Nagumo neurons but fairly different and worse for the Hodgkin-Huxley neurons. Indeed, the sensitivity is much higher for the latter and the derivation is only valid when the populations are weakly connected, as shown in Figure 6 bottom middle and right.

The left picture in Figure 7 shows a small variation of the distance between the full network and the reduced system. Indeed, even the strongest variation for these parameters is one order of magnitude smaller than the typical variation of the signal in Figure 6 top middle. This suggests the reduction is robust to the variation of these parameters within the reasonable range of values chosen.

However, the right picture shows that the slowness of the synapses is a critical feature enabling the reduction. The synapses have to be at least one order of magnitude slower than the neurons' activity. Yet, the fastest synapses enabling the reduction, i.e. 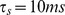, are reasonably fast with respect to measurements. Thus, this restriction is relevant and the reduction can be performed in biological regimes.

## Discussion

Even if collective phenomena arising in large noisy spiking neural networks are extremely complex, we have shown that, under some assumptions and for some models, a macroscopic variable describing the global behavior of the network can be consistently described by simple low dimensional deterministic differential equations. The parameters and non-linearity involved are determined by the type of neurons considered and by the level of noise neurons are subjected to. Depending on the neuron model the non-linearity can be a simple, well-behaved function (which we call regime I) or a more complicated multivalued function (which we call regime II in case of two values). The three neuron models we considered (McKean, Fitzhugh-Nagumo and Hodgkin-Huxley) are regime I when the intrinsic noise is strong in the network, and we expect this property to be valid for any type of neuron models. However, with weak noise the Hodgkin-Huxley model is regime II, in which case the low-dimensional model proposed is more complicated (involving jumps between attractors). Comparisons of the averaged dynamics of spiking networks with the reduced equations showed a very precise fit, even for initial conditions independent of the network initial conditions, for regime I neuron models. However, for the regime II neuron models, the reduction accuracy is not as good. Indeed, noise will induce random switches from one attractor to the other, which cannot be handled through reduced methods, and therefore path-wise fit are bound to be out of reach. Yet, the reduced model recovers the main qualitative features of the signal, but in the bistable regions, quantitative distinctions arise.

For neurons in regime I, the reduction accuracy is significantly better for McKean and Fitzhugh-Nagumo neuron models than for the Hodgkin-Huxley model. Indeed, the reduction for the latter becomes irrelevant for strong connections between neurons whereas it is not the case for the former. We believe this is not due an inherent difference between the models, but rather to an inadequacy of the choice of the linear part 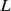 for the Hodgkin-Huxley model in such parameter regimes. Indeed, as shown in table 1, there is some time-delayed information in the linear part of McKean and Fitzhugh-Nagumo whereas there is simply a linear instantaneous decay for Hodgkin-Huxley model. Analyzing the origin of this memory and the adequate term to be considered in the Hodgkin-Huxley networks is an exciting problem that we are currently investigating.

This reduction relies on a number of assumptions imposed by the mathematical approach: first, the approach is valid when neuronal populations are large and randomly connected for averaging effects to occur (i.e. for the mean-field reduction to hold). More importantly, the reduction is largely based on the linearity of synapses. As said in the introduction, this assumption is not fully consistent with the biological system. It was however necessary to perform the reduction. Extending this approach to non-linear synapses is an important improvement to increase biological plausibility of these results well worth investigating. Another assumption was the slowness of synapses and inputs. This assumption, required in our mathematical developments, does not seem critical. Indeed, simulations have shown that the reduction was quite robust to increased speed for synapse and inputs.

It is important to note the extreme complexity reduction obtained: in the case of Fitzhugh-Nagumo networks, we reduced a system of 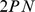 stochastic differential equations with 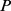 populations into a system of 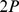 deterministic, ordinary one dimensional differential equations. The nonlinear transforms computed, as well as code for the simulations, are freely provided online. For efficient simulation of large-scale neuronal spiking networks with noise, if one is interested in computing the mean macroscopic activity, simulating the reduced model is a precise and simple choice that shall be considered for efficiency.

This study quantifies the stabilization properties of the noise, that were already discussed in [34], controlling the shape of the effective non-linearity of the reduced model. Noise tends to act as a linearizer: when the noise is strong, the network macroscopic activity tend to evolve more linearly. The example of Hodgkin-Huxley model shows it can even change a neuron model from regime II to regime I. This implies that knowing the value of the intrinsic noise in biological tissues could be a good indicator to evaluate their level of non-linearity.

As opposed to former reduction techniques [25], we have presented here a way to reduce networks of large populations of neurons to ordinary differential equation reminiscent to the heuristically motivated Wilson-Cowan equations [12]. However, a notable difference with these Heuristic models is the time delayed feature of the linear part of these reduced equations, corresponding to the adaptation variable. This motivates the study of classical neural networks with adaptation which could be an important feature of information processing in the brain.
